# Sea lice exposure to non-lethal levels of emamectin benzoate after treatments: a potential risk factor for drug resistance

**DOI:** 10.1038/s41598-020-57594-7

**Published:** 2020-01-22

**Authors:** Chun Ting Lam, Sarah M. Rosanowski, Martin Walker, Sophie St-Hilaire

**Affiliations:** 1 Department of Infectious Diseases and Public Health, Jockey Club College of Veterinary Medicine and Life Sciences, City University of Hong Kong, Kowloon Tong, Hong Kong SAR PRC; 2 Department of Pathobiology and Population Sciences, Royal Veterinary College, University of London, Hatfield, UK

**Keywords:** Parasitic infection, Antimicrobial resistance

## Abstract

The avermectin derivative emamectin benzoate (EMB) has been widely used by salmon industries around the world to control sea lice infestations. Resistance to this anti-parasitic drug is also commonly reported in these industries. The objective of this study was to quantify the number of sea lice potentially exposed to sub-lethal concentrations of EMB while fish clear the drug after treatments. We assessed juvenile sea lice abundance after 38 EMB treatments on six Atlantic salmon farms, in a small archipelago in British Colombia, Canada, between 2007 and 2018. We fitted a standard EMB pharmacokinetic curve to determine the time when fish treated with this product would have EMB tissue concentrations below the recommended target therapeutic level. During the study, we estimated that for each sea lice treatment there was, on average, an abundance of 0.12 juvenile sea lice per fish during the time period when the concentrations of EMB would have been lower than 60ppb, the recommended therapeutic treatment level for sea lice. The findings from this study on metaphylactic anti-parasitic treatments identify a potential driver for drug resistance in sea lice that should be further explored.

## Introduction

Sea lice, specifically *Lepeophtheirus salmonis* and *Caligus spp*., is a group of grossly visible host-dependent ectoparasites^1,2^ commonly found in all large salmonid aquaculture industries around the world. Treatment of these parasites use chemical baths and/or in-feed pharmaceutical products such as the avermectin derivative emamectin benzoate (EMB)^3–5^. In the case of EMB, most salmonid aquaculture industries globally, with the possible exception of the industry in British Columbia (BC), Canada, have documented the emergence of EMB resistant sea lice within 5 to 10 years of the product’s introduction^3,4,5,6,7^.

The U.S. Food and Drug Administration proposes several management strategies that may increase the risk of anti-parasitic resistance in veterinary medicine, including overuse and under dosing of products^8^. Both of these phenomena occur in salmon industries, as there are few products licensed for sea lice treatments; hence, overuse of single products is inevitable. Further, under-dosing of sub-groups of fish within a population has been documented during metaphylactic treatments on salmon farms^9^. Sea lice exposure to sub-lethal levels of EMB may also occur if fish are re-infected with parasites shortly after treatment, while clearing the drug. Given the dynamics of sea lice infestations within salmon farming areas^10^, and the size of farm populations treated, the selection pressure for drug resistant sea lice may be significant. Despite this there are no studies, which have assessed the level of re-infection during the period when fish would have sub-lethal levels of EMB in their tissues.

All large salmon industries have been monitoring sea lice abundance by life stage on individual farms for over a decade, which provides researchers the opportunity to evaluate the re-infection pressure after different types of anti-parasitic treatments. In light of the urgent need to better understand the impact of different treatment strategies on emerging anti-parasitic drug resistance, and the need to increase the life span of new, in-feed anti-parasitic treatments, we explored sea lice infection after metaphylactic treatments with EMB. Specifically, we determined the re-infection pressure after oral (in-feed) EMB treatments on salmon farms, during the period when animals were clearing the anti-parasitic medication and were likely below the EMB manufacturer’s recommended therapeutic threshold.

## Methods

Sea lice abundance data from six commercial Atlantic Salmon (*Salmo salar*) farms in a small archipelago in British Columbia, Canada, for the period from January 2007 to January 2018, were extracted from the BC Salmon Farmers Association database. Sea lice counts were performed by farmers as part of the routine assessment required by the Department of Fisheries and Oceans, Canada^11^,^12^. Each farm reported the number of juvenile (Chalimus I&II) and mobile sea lice. Mobile sea lice were reported separately for *Caligus sp*. and *Lepeophtheirus salmonis,* but the different types of juvenile lice were not differentiated. We used the counts for juvenile sea lice and for adult mobile  *L. salmonis* lice in this study. Counts were conducted bi-weekly or monthly, depending on the time of the year. The farmers also included the total number of fish sampled, so we were able to calculate the abundance of juvenile and adult (mobile) sea lice per fish for each farm.

We also obtained the total number of fish on the farms for each month, the daily water temperature, and the dates when emamectin benzoate (EMB) treatments were administered. To determine the number of fish on the farm for each week, we assumed the fish losses over the month were evenly distributed across the weeks, and used this value to estimate the total number of sea lice on the farm (i.e. abundance × total number of fish).

For all 40 EMB treatments in the 11-year study period dataset we extracted the sea lice data 1 week prior to and up to 10 weeks post EMB treatments. Thirty-three of the treatments were standard applications of EMB, lasting between 7 and 10 days in duration, at a dose of 50 µg/kg of fish. The other seven treatments were either shorter than 7 days or longer than 10 days. Based on the manufacturer’s instructions, the standard EMB treatment regime (i.e. 7 days at 50 µg/kg) is sufficient to achieve the manufacturer’s recommended target therapeutic dose of 60 ppb in muscle and skin^13^. Previously published bioassay studies suggest that, *in vitro*, higher concentrations than 60 ppb may be required for 100% efficacy^14^,^15^.

**Figure 1:**
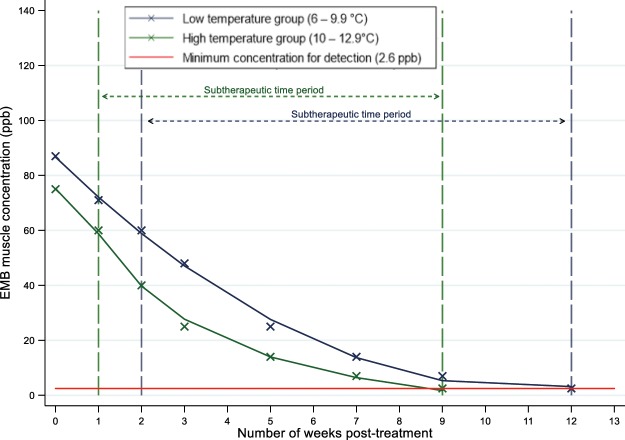
Derived emamectin benzoate residue depletion curve for salmon muscle tissue based on laboratory studies conducted by Glover *et al*. (2010), Roy *et al*. (2006), Sevatdal *et al*. (2005), and Skilbrei *et al*. (2008) at different water temperatures^16–19^. The green and blue solid lines indicate the two EMB pharmacokinetic curves used for this study, fitted with a fractional polynomial curve. The green and blue dash lines indicate the time period when fish were considered to have “sub-therapeutic” or non-lethal concentrations of EMB in their tissues for the two categories of water temperatures. The red line indicates the limit of detection (2.6 ppb) of EMB in muscle tissue^39^.

To establish the time period when fish would have had detectable concentrations of EMB in their tissues above (and below) the recommended therapeutic threshold concentration, we applied one of two extrapolated pharmacokinetic curves for EMB, from four published laboratory studies conducted at various water temperatures^16,17–19^ (Fig. 1). The curves were created by fitting a fractional polynomial curve to the published data. Each treatment was categorized into one of two groups, based on the water temperature at the end of the treatment period: a low water temperature group (6–9.9 °C) and high water temperature group (10–12.9 °C). It was estimated that levels of EMB > 60 ppb in fish lasted approximately 1 week from the last day of treatment for fish in the high water temperature group, and 2 weeks for fish in the low water temperature group. After the 1 or 2 week period , we assumed, based on laboratory studies, that tissue concentrations of EMB were below recommended therapeutic levels; therefore, we refer to them as “sub-therapeutic” or “sub-lethal”. The period of time when new lice were exposed to lower concentrations of EMB than the recommended target therapeutic level was estimated to last 8 weeks for the high water temperature groups and 10 weeks for the low temperature groups^16,17–19^. Beyond 12 weeks, regardless of temperature, EMB was no longer detectable in any of the pharmacokinetic studies used to inform our study^20^. It could be argued that the therapeutic level of EMB for sea lice is below 60 ppb, so we also determined the sea lice re-infection rate when EMB concentrations would theoretically be below 40 ppb as a conservative comparison. At the higher temperature range, the time window of interest for the 60 ppb and 40 ppb cut offs were 8 and 7 weeks, respectively (Fig. 1). In both EMB threshold assessments, we used the maximum juvenile sea lice count reported during the sub-therapeutic period to estimate sub-lethal exposure to EMB. To obtain the total exposure for the study period, we summed the maximum sea lice count during this period for each of the treatments. We focused on the juvenile sea lice because they are indicative of new infections.

We also estimated the efficacy for EMB treatments by subtracting the average adult *L. salmonis* abundance over three weeks post treatment from the abundance one week before the treatment, and divided this number by the latter. We used an average value over a three-week period because there were numerous weeks of missing data during the study period. We decided to use a three-week window instead of two weeks to maximize the number of treatments for which we could estimate efficacy. We did not include more than 4 weeks of data post treatment for detemining efficacy of the treatment because we did not want to count adult sea lice that had possibly originated from new infections post treatment, given the average water temperature in our study^21,22^.

## Results

There was a total of 40 EMB treatments during our study period, but only 38 treatments had sea lice data for the time period when fish would have had EMB levels below 60 ppb. Water temperatures during these treatments ranged between 4.0 °C and 14.5 °C, with an average temperature of 9.1 °C at the end of the sea lice treatments (Table 1). The number of fish on the six farms in our study ranged from 260,968 to 947,384 (average 551,742) (Table 2). Over the course of this 11 year study, given the size of farms and the sea lice abundance recorded, millions of adult and juvenile sea lice were exposed to the target therapeutic levels of EMB (Tables 2 and 3). The efficacy of the treatments could only be measured for 17 treatments, due to missing sea lice count data either before or after treatment. Of these treatments, only 30% (5/17) had a reduction in adult sea lice of 80% or more.

**Table 1:** Descriptive statistics of the duration of emamectin benzoate (EMB) treatment and water temperature on six commercial Atlantic Salmon (Salmo salar) farms in a small archipelago in British Columbia, Canada, between January 2007 and January 2018.

Variables	Number of treatments with observations	Mean	Standard deviation	Range	
				Min	Max
Duration of EMB treatments (days)					
Standard treatments	33	8.00	1.06	7	10
Non-standard treatments	7	11.14	6.77	2	20
Overall	40	8.55	3.07	2	20
Water temperature (°C) at the end of EMB treatment					
Overall	40	9.06	2.04	6.00	13.04

**Table 2:** Estimation of fish numbers on farms and sea lice abundance exposed to therapeutic (≥60 ppb) and sub-therapeutic (<60 ppb) concentrations of emamectin benzoate (EMB) after treatments. Data collected by farmers on six commercial Atlantic Salmon (*Salmo salar*) farms in an archipelago in British Columbia, Canada, between January 2007 and January 2018.

Variables	Number of treatments with observations	Mean	Standard deviation	Range	
				Min	Max
Salmon population number in the study area					
In Spring (April - June)	8	622,084	142,728	482,844	942,137
In Summer (July - September)	7	466,548	90,410	342,904	582,821
In Fall (October - December)	17	543,902	134,325	260,968	795,729
In Winter (January - March)	8	572,604	170,370	390,178	947,384
Overall	40	551,742	141,279	260,968	947,384
Abundance of adult sea lice exposed to therapeutic level (≥60 ppb) of EMB					
In Spring (April - June)	3	3.75	3.77	1.43	8.09
In Summer (July - September)	3	8.08	13.39	0.27	23.54
In Fall (October - December)	10	8.79	11.39	0.05	35.10
In Winter (January - March)	4	3.44	4.30	0.32	9.70
Overall	20	6.86	9.54	0.05	35.10
Abundance of adult sea lice exposed to sub-therapeutic level (<60 ppb) of EMB					
In Spring (April - June)	7	4.13	4.21	0.72	12.90
In Summer (July - September)	7	3.61	4.35	0.03	9.40
In Fall (October - December)	16	3.05	3.86	0.10	12.26
In Winter (January - March)	8	2.29	2.90	0.12	9.02
Overall	38	3.19	3.74	0.03	12.90
Abundance of juvenile sea lice exposed to therapeutic level (≥60 ppb) of EMB					
In Spring (April - June)	3	0.04	0.04	0	0.08
In Summer (July - September)	3	0.26	0.18	0.13	0.47
In Fall (October - December)	10	0.40	1.12	0	3.58
In Winter (January - March)	4	0.07	0.09	0	0.2
Overall	20	0.26	0.79	0	3.58
Abundance of juvenile sea lice exposed to sub-therapeutic level (<60 ppb) of EMB					
In Spring (April - June)	7	0.04	0.03	0	0.08
In Summer (July - September)	7	0.41	0.40	0	1.13
In Fall (October - December)	16	0.08	0.11	0	0.35
In Winter (January - March)	8	0.05	0.10	0	0.30
Overall	38	0.12	0.23	0	1.13

**Table 3:** Estimation of sea lice abundance exposed to therapeutic (≥40 ppb) and sub-therapeutic (<40 ppb) concentrations of emamectin benzoate (EMB) after treatments. Data collected by farmers on six commercial Atlantic Salmon (*Salmo salar*) farms in an archipelago in British Columbia, Canada, between January 2007 and January 2018.

Variables	Number of treatments with observations	Mean	Standard deviation	Range	
				Min	Max
Abundance of adult sea lice exposed to therapeutic level (≥40 ppb) of EMB					
In Spring (April - June)	4	3.25	3.23	1.43	8.09
In Summer (July - September)	3	8.08	13.39	0.27	23.54
In Fall (October - December)	16	6.71	9.60	0.05	35.10
In Winter (January - March)	7	2.67	3.24	0.12	9.70
Overall	30	5.44	8.21	0.05	35.10
Abundance of adult sea lice exposed to sub-therapeutic level (<40 ppb) of EMB					
In Spring (April - June)	7	4.13	4.21	0.72	12.90
In Summer (July - September)	7	3.55	4.40	0.02	9.40
In Fall (October - December)	15	2.65	3.93	0.10	12.17
In Winter (January - March)	7	1.87	3.30	0.10	9.02
Overall	36	2.96	3.88	0.02	12.90
Abundance of juvenile sea lice exposed to therapeutic level (≥40 ppb) of EMB					
In Spring (April - June)	4	0.05	0.04	0	0.08
In Summer (July - September)	3	0.42	0.26	0.13	0.65
In Fall (October - December)	16	0.29	0.88	0	3.58
In Winter (January - March)	7	0.09	0.11	0	0.30
Overall	30	0.22	0.65	0	3.58
Abundance of juvenile sea lice exposed to sub-therapeutic level (<40 ppb) of EMB					
In Spring (April - June)	7	0.04	0.03	0	0.07
In Summer (July - September)	7	0.32	0.44	0	1.13
In Fall (October - December)	15	0.05	0.10	0	0.35
In Winter (January - March)	7	0.01	0.02	0	0.07
Overall	36	0.09	0.23	0	1.13

We estimated that over a period of 11 years the juvenile sea lice abundance on farms ranged between 0 and 1.13 when fish would have had levels of EMB below the 60 ppb threshold (Table 2). The range of juvenile sea lice abundance exposed to sub-lethal EMB levels remained the same (i.e. 0 to 1.13) when we lowered the therapeutic threshold to 40 ppb, but on average, there were fewer lice exposed to EMB associated with this threshold cut-off (Tables 2 and 3). The average numbers of lice exposed, post treatment, to EMB levels below 60 ppb and 40 ppb for treatments administered in the summer, when the infection rate was highest, were 41 and 32 juvenile sea lice per 100 fish, respectively (Tables 2 and 3).

## Discussion

Our study identified a possible issue with the medicinal application of an anti-parasitic drug that may increase the risk of resistance: exposure of parasites to “sub-therapeutic” or sub-lethal drug concentrations after treatment. Using a salmon sea lice model with extensive surveillance data, we determined that sea lice infection pressure on salmon farms exists post lice treatments, and fish treated with EMB were re-infected with juvenile sea lice while they were clearing the medication. This would have exposed sea lice in the area to sub-lethal levels of EMB. We hypothesize that this may be one of the reasons why sea lice develop resistance to EMB. This phenomenon is similar in concept to the use of low dose antimicrobials for growth promotion, which has also been associated with bacterial antibiotic resistance^23–29^.

The extent and duration of exposure to low concentrations of an anti-parasitic drug after a treatment depends on the half-life of the medication used, the metabolic rate of the host, the size of the animal population treated, and the pathogen (re-)infection pressure on the host. When treating large populations of animals, the post-treatment exposure to sub-lethal levels of medication may be significant, especially for pharmaceuticals that have a long half-life and are not quickly cleared by the animals. To estimate the scale of this issue on large fish farms, we used EMB treatments and sea lice surveillance data over an 11-year period and applied published pharmacokinetic data for EMB to establish the temporal window when fish would have had sub-lethal levels of drugs in their tissues. We found, even in a fish farming area such as British Columbia, with relatively low sea lice infestation rates, that juvenile sea lice were exposed to sub-lethal levels of EMB, as they were detected on fish during the window of time when fish were clearing the medication.

The sensitivity of sea lice to EMB varies within and between sea lice populations^14^,^15^, so the 60 ppb threshold used in this study may not reflect the precise lethal concentration for 100% of the population, but it is the recommended target level by the manufacturer^13^. We also explored lowering the threshold to 40 ppb, which shortened our time window by up to 2 weeks. Despite this change, we still detected juvenile sea lice when fish presumably had EMB at concentrations above the limit of detection but below 40 ppb (Table 3).

The abundance of juvenile sea lice on fish post treatment was very low in this study (i.e. range of 0.14 to 0.41 juvenile lice per fish, depending on the season), but given the number of fish on farms and the number of farms within the industry, the number of juvenile lice exposed to non-lethal levels of EMB could be high over time. In other salmon farming areas around the world with higher levels of sea lice infestations and higher densities of fish farms, this phenomenon may be a significant driver of sea lice drug resistance. Low dose exposure to EMB may select sea lice with a genetic predisposition to full or partial resistance to the product^28^. In bacterial populations, the mechanism for selection of antibiotic resistance associated with sub-lethal levels of drugs is likely multifold^28^. Exposure of bacterial populations to low level antibiotics can select for existing antimicrobial resistance, as well as induce protective mechanisms within these populations, which includes an increase in the mutation rate of the organisms^30,31^. The latter enables bacteria to more rapidly adapt to their environment^30,31^. Whether this is the case with sea lice is unknown.

Given the limited number of anti-parasitic products available to treat sea lice, it is important to increase the length of time that each medication is effective. Preventing re-infection with sea lice, especially during the period when fish may have sub-lethal levels of EMB, may be an important management strategy for reducing the risk of developing EMB-resistant sea lice. Preventing re-infection during this period requires an understanding of the sources of sea lice for fish on farms. In our study, there were likely two primary sources of sea lice: returning wild fish infested with adult sea lice^32,33^ and infected farm fish within the area, including the farm in question and its neighbors^34–36^.

In British Colombia, the number of migrating (wild) fish through salmon rearing areas varies, but can be in the millions. The seasonal source of “wild-type” sea lice from wild adult salmon migration through the farming areas in the summer and autumn period likely provides a natural pool of EMB- naïve lice annually, which may reduce the risk of selecting for resistant sea lice^32,37^. However, if the industry exposes these lice to low doses of EMB they may lose the beneficial effect of this natural parasite “refugia”. While infection resulting from returning wild adult salmon cannot be avoided, this source of infection is limited to a short period of time and is very predictable. Treatment applications on farms during or before the wild adult salmon migration period may increase the probability of becoming re-infected after treatment and exposing sea lice to sub-lethal EMB concentrations. To reduce the likelihood of re-infection after treatment, during the migratory period of the year, it may be best to delay sea lice treatments, especially if they are not administered for clinical reasons, until all returning adult wild fish have migrated through the area.

Controlling re-infection of sea lice from farm fish sources is complicated, as this source of infection is not seasonal. It is possible that juvenile sea lice on fish in this study came from adult lice on fish within the farm, as treatment efficacy appeared to be quite poor. The reason for the apparent treatment failure could not be determined in this study and could have been due to several issues. For example, the actual dose of EMB delivered to the fish may not have been sufficiently high to treat all adult life stages. It is also possible that the method of determining treatment efficacy in this study was inaccurate, as we used an average number of lice over three weeks post treatment. Lastly, the sea lice may be starting to develop resistance to the product. Regardless if the new juvenile sea lice infections were from adults that survived EMB, the fact that they were present when the fish were clearing the medication could exacerbate the resistance issue by selecting for lice that are more tolerant of EMB. This phenomenon may explain why sea lice resistance to EMB occurs within 5 to 10 years of use, as has been seen in many salmon industries^3,4,5,6,7^ that have used this product more frequently than the BC industry.

It is also possible that new post-treatment infections originated from neighbouring farms in the area, as sea lice transmission between farms can occur over several kilometres^10,35^. To prevent re-infection during the (high risk) period, when fish are clearing EMB and tissue concentrations fall below the recommended therapeutic levels for maximum efficacy, farmers could apply a chemical bath treatment as soon as juvenile sea lice are detected on fish. Chemical bath treatments are effective; however, their effect is temporary (i.e. there is no significant residual effect after the bath is completed). Therefore, frequent applications may be required to prevent re-infection if the infection pressure is continuous during the time when fish are clearing EMB from their tissues^38^. In British Colombia, the only chemical bath product that is licensed for use is hydrogen peroxide, and its efficacy against juvenile stages of sea lice is poor^41^. In the case that juvenile lice cannot be effectively removed, it may be necessary to treat fish with a chemical bath after the juvenile lice stages have developed into the pre-adult stage, before the lice start to reproduce, to mitigate any selection that may have occurred from exposure to low concentrations of EMB. This treatment time window would vary depending on the water temperature but, regardless, it would be quite short and require that the industry monitor juvenile sea lice more closely. In most salmon industries, the decision to treat for sea lice is made based on adult sea lice counts; therefore, to implement a management strategy based on juvenile lice counts would require a significant change in practices.

In cases where the source of infection is neighbouring farmed fish, it may also be possible to reduce re-infection by synchronizing treatments on farms in an area. This has been shown to reduce the overall lice burden in Chilean farms^39^; however, in areas where there are many fish farms, synchronization of sea lice treatments poses logistical difficulties. It may also lead to excessive use of anti-parasitic products, which could increase the likelihood of resistance^40^. The difficulty in preventing re-infection with sea lice shortly after in-feed treatments on salmon farms highlights the urgent need for alternative effective non-chemical treatments for this parasite.

Our study was the first to apply an EMB pharmacokinetic curve to determine the post-treatment period when fish are likely to have low concentrations of this drug in their skin, which could select for EMB resistant sea lice. Infection pressure during the period after a treatment is seldom taken into consideration as a risk factor for resistance, but if animals are re-infected shortly after treatment it may be important. One of the limitations of our study was that it was based on retrospective sea lice data collected at the farm level, and we lacked empirical pharmacological information on our treated fish populations. Under field conditions, where medication is delivered as in-feed metaphylactic treatments, tissue concentrations of EMB can vary between fish^10^, hence our “sub-therapeutic” drug exposure period post-treatment (based on laboratory data) may not reflect precisely the risk period. If fish were under-dosed during their treatments, then the estimated “sub-therapeutic” period would commence earlier and end sooner, and if fish received more than the expected dose the window would extend beyond the 8 or 10-week period estimated in this study.

Another limitation of our study was that farmers were not obligated to collect weekly sea lice counts, so the estimate of the number of new lice exposed to non-lethal levels of EMB was not precise. In some cases, counts were taken bi-weekly or monthly, so we used the maximum count of sea lice during the window to indicate exposure. Given that juvenile sea lice can continuously infect fish and mature into pre-adult mobile lice within 3 weeks^21,22^, it is possible the exposure to juvenile lice was higher than estimated in this study. Empirical data on EMB concentrations in fish populations post-treatment and weekly sea lice re-infection measurements during this window would enable us to validate our study findings. These data would allow more accurate determination of the duration of exposure to low drug concentrations, as well as the sea lice re-infection pressure during this time frame.

Regardless of the uncertainty in our data, our study demonstrated that lice exposure to low concentrations of EMB is inevitable after treatment. Even within this relatively isolated population of fish, and using our conservative estimate for the window during which fish metabolize EMB, we were able to identify re-infections with new lice. In countries with larger farming areas and with more extensive use of EMB, exposure to low concentrations of this drug would be significantly greater.

As open net-pen aquaculture systems increase in size, farms become more interconnected and the potential for sharing pathogens increases, the need for antimicrobials and anti-parasitic drugs to control disease outbreaks also increases, so it becomes more important to understand and minimize the impact of medical practices on the development of drug resistance. In the broader context, our results demonstrate that the likelihood of exposure to “sub-therapeutic” drug concentrations is high in large popualtions when animals are metabolizing or clearing medication from their systems. Although this study was conducted on a fish parasite system, it highlights an issue that may be important for curtailing the development of drug resistance during population treatments in other animal species, where rapid re-infection is inevitable. Further studies are needed to investigate whether this phenomenon is also a driver for drug resistance in bacterial populations.

## Data Availability

Data was obtained from the BC Salmon Farmers Association. We are not able to share this information due to a confidentiality agreement with the company that owns the data.
